# No significant HTLV seroprevalence in German people who inject drugs

**DOI:** 10.1371/journal.pone.0183496

**Published:** 2017-08-22

**Authors:** Oliver Hohn, Stephen Norley, Claudia Kücherer, Ali Bazarbachi, Hiba El Hajj, Ulrich Marcus, Ruth Zimmermann, Norbert Bannert

**Affiliations:** 1 Department of Infectious Diseases, Division for HIV and Other Retroviruses, Robert Koch Institute, Berlin, Germany; 2 Department of Internal Medicine, Department of Cell Biology, Anatomy and Physiological Sciences, American University of Beirut, Beirut, Lebanon; 3 Department of Internal Medicine, Department of Experimental Pathology, Microbiology and Immunology, American University of Beirut, Beirut, Lebanon; 4 Department for Infectious Disease Epidemiology, Division for HIV/AIDS, STI and Blood-borne Infections, Robert Koch Institute Berlin, Germany; Centers for Disease Control and Prevention, UNITED STATES

## Abstract

**Background:**

Although human T-lymphotropic virus (HTLV) is transmitted via the same routes as human immunodeficiency virus (HIV), its worldwide seroprevalence differs drastically because HTLV is transmitted mainly via infected cells rather than free virus. The sharing of needles and other equipment places people who inject drugs (PWID) at particularly high-risk for such blood-borne diseases.

**Methods:**

To validate the methodology used to process and analyze the dried blood spots (DBS) utilized in the study, dried serum spots (DSS) with dilutions of sera from known HTLV infected individuals were analyzed by ELISA and Western blot. DBS collected between 2011 and 2015 from 2,077 PWID in eight German cities recruited by respondent-driven sampling were tested for HTLV-specific antibodies.

**Results:**

The validation demonstrated that the use of DSS allowed identification of samples with even low titers of HTLV-specific antibodies, although a confirmatory Western blot with an additional venous blood sample would often be required. Despite numerous HIV and HCV positive individuals being identified within the study population, none tested positive for HTLV.

**Conclusion:**

While the HIV and HCV prevalences in German PWID are comparable to those in other European countries, the very low prevalence of HTLV reflects the situation in the general population.

## Introduction

Human T-lymphotropic virus (HTLV) was the first exogenous human retrovirus discovered [1], and although three additional types of HTLV have since been described, only HTLV type 1 is found throughout the world [2–4]. HTLV originated by transmission of simian T-lymphotropic viruses from non-human primates to humans, but only HTLV-1 and HTLV-2 are so far known to be transmitted from human to human [5–8]. Although HTLV-1 can be further divided into seven subtypes (A-G), only the cosmopolitan subtype A is found world-wide [9, 10]. There is, as yet, very little data concerning the distribution of HTLV-2, a virus found in several native populations in North and South America and in pygmy tribes of Central Africa [11, 12]. However, HTLV-2 infection of people who inject drugs (PWID) [12] suggests that this distribution is now broadening globally. HTLV types 3 and 4 have only so far been found in a few individuals in Africa [6, 13, 14]. HTLV-1 is the causative agent of adult T-cell leukemia/lymphoma (ATL) [15], HTLV-1-associated myelopathy/tropical spastic paraparesis (HAM/TSP) [16] and HTLV-1-associated uveitis (HAU) [17]. HTLV-1 is also thought to cause local and systemic inflammation which, in addition to HAM/TSP for which the most data is available, probably leads to several other diseases collectively referred to as HTLV-1 associated inflammatory diseases (HAID) [18]. Data concerning HTLV-2 infection and diseases remains limited, but HTLV-2 also seems to be associated with HAM/TSP [12, 19] and other neurological disorders [12, 20] although the association with increased mortality from cancer remains controversial [21, 22]. A higher incidence of bladder or kidney infection and arthritis has been observed for both HTLV-1 and HTLV-2 infection, and HTLV-2 infected individuals have been shown to have a higher incidence of acute bronchitis and pneumonia than HTLV-seronegative individuals [22].

There are three well-studied routes of human-to-human transmission for both HTLV-1 and HTLV-2: sexual contact, breastfeeding and blood products containing HTLV infected lymphocytes [10, 12]. Despite the fact that these routes of transmission resemble those for HIV-1, the global distribution of HTLV-1 is strikingly different. It is estimated that world-wide, 5–10 million people are currently infected with HTLV-1, but these are mainly concentrated in just a few areas of high endemicity such as sub-Saharan Africa, South America and the Southwestern region of Japan [10]. Furthermore, clusters exist in the Caribbean and the Middle East, but near these regions of high prevalence there are areas with low endemicity. Within Europe, Romania is a country of relatively high HTLV-1 prevalence, with 5.33 of 10,000 first time blood donors testing positive [23]. The reasons for this unbalanced distribution are not yet fully understood, but founder effect and the persistence of virus transmission by one of the three major routes seems likely. For example, Japanese programs to prevent mother-to-child transmission via breast-feeding resulted in a marked reduction of HTLV transmission [24].

The storage and shipping of samples for subsequent detection of antibodies in epidemiological surveys can be simplified if dried blood spots (DBS) or dried serum spots (DSS) on filter paper are used. Numerous studies investigating various pathogens have shown DBS to be reliable for antibody detection and comparable to venous blood samples. DBS were successfully evaluated soon after the start of the HIV/AIDS pandemic and the method has since proven to be very useful for HIV-specific antibody screening [25, 26]. DBS were shown to be reliable for detecting anti-HIV-1/HIV-2 antibodies and serological markers of other pathogens, using either in-house or commercial assays to analyze the eluates [27, 28]. The use of DBS in the detection of anti-HTLV antibodies has also been validated in several studies [29–31]. One early study initially established the methodology using simulated DBS, (i.e. known HTLV-positive serum (or plasma) samples diluted in HTLV-negative human blood cells) and then screened 10,135 DBS from neonates using the HTLV-1 gelatin particle agglutination test, confirming by ELISA and Western blot [31]. An ELISA followed by Western blot is still recommended for HTLV diagnosis by the HTLV European network (HERN) [32]. Using this procedure, a panel of 23 serum samples provided by HERN and subsequently 126,010 DBS samples from pregnant women in the United Kingdom were tested for the presence of HTLV antibodies. HTLV antibodies could be reliably detected even in dried blood spot eluates derived from patients with serum titres as low as 1:50 in a modified anti-HTLV gelatin particle agglutination assay [33]. In addition, several HTLV seroprevalence studies have been performed using DBS/DSS but without method validation [34–36]. According to the available sparse data, the prevalence of HTLV infection in Germany within the overall population is very low. The rate among 58,747 neonates delivered in Berlin between 1996 and 1997 and screened by heel prick was found to be only 0.7 in 10,000 DBS, the lowest in Europe [35], although a screening of 248,000 sera from blood donors in the early 90's found a prevalence of 2.1 in 10,000 [37]. However, in addition to all German studies so far being somewhat out of date, only one included people with a known risk for blood-borne diseases (although relatively few). In addition to 100,852 samples from blood donors, this Frankfurt study screened 117 from hemophiliacs and 63 from local PWID but failed to identify a single, confirmed case of HTLV-1 infection [38].

The first aim of the study presented here was to validate, in our hands, the use of DBS for HTLV testing using a small set of sera from known HTLV-positive individuals. The second was to determine the prevalence of HTLV in a German cohort of people who inject drugs (and therefore at high risk for blood-borne infections) using 2,077 DBS from PWID in eight German cities that had been collected using respondent-driven sampling as part of a multicenter sero-behavioural survey for HIV and hepatitis B and C [39].

## Materials and methods

### Serological testing of DBS

As part of a recent study using respondent-driven sampling, 2,077 DBS from PWID in eight German cities had been collected, with the number of DBS in the respective cities ranging from 130 (Leipzig) to 337 (Berlin). The capillary blood samples, collected by a trained person, were spotted with ~30μl per spot on filter cards (Whatman 903, GE Healthcare Life Sciences, Freiburg, Germany). The filter cards were dried for at least three hours or overnight at room temperature, then placed in plastic bags and sent to our laboratory. Antibodies were eluted in PBS containing 0.05% Tween-20 and 3% FCS by incubation overnight at 4°C, resulting in a 1:15 dilution with respect to the original blood volume. Eluates were stored at -20°C until testing for the presence of HTLV-specific antibodies according to a modified algorithm proposed by the HTLV European Research Network [32]. First, eluates were tested individually with the “Murex HTLV I+II ELISA” (Diasorin, Dietzenbach, Germany) according to the manufacturer's instructions. Positive samples were tested again in duplicate with the same assay. For repeatedly positive samples, the intention was to contact the donor for a follow-up venous blood sample which would be used to repeat the ELISA and confirm seropositivity using the Western blot assay “MP Diagnostics HTLV Blot 2.4” (MP Biomedicals SAS, Illkirch Cedex, France) according to the manufacturer's instructions. If the participant was not available for a follow-up sample then stored DBS material from this person could have been used to confirm HTLV infection via PCR as described elsewhere [40].

### Validation of HTLV testing

To validate HTLV testing from DBS, 14 randomly selected sera from individuals previously tested positive for HTLV antibodies were used. All subjects were asymptomatic carriers (AC) to reflect the expected situation within a group of non-hospitalized PWID. Characterized HTLV-1 and HTLV-2 sera for the Dried Blood Spot validation were additionally provided by the Communicable Diseases Research Tissue Bank of Imperial College Healthcare NHS Trust, London, UK. The proviral loads of these HTLV infected subjects ranged between 0.01% and 35%. Thirty μl of the undiluted sera as well as of three serial 10-fold dilutions in negative human sera were spotted onto two replicate filter papers. These DSS were then processed in the same way as the DBS, eluting the antibodies from the filters to give an additional 1:15 dilution before testing in ELISA. A confirmation Western blot of the highest dilution of each sample still reactive in ELISA was carried out as described above.

### Determination of HTLV prevalence among PWID in Germany

Details of the study protocol have been published elsewhere [39, 41]. Briefly, between 2011 and 2014 a sero-behavioural survey was conducted among PWID in eight German cities in cooperation with low-threshold drug services to generate HBV-, HCV- and HIV- seroprevalence- and associated behavioral data among current injectors (DRUCK-study). Respondent-driven sampling was used to reach PWID who were not in regular contact with the low-threshold drop-in facilities or drug consumption rooms but were part of the social network of other participants. Inclusion criteria for the survey were (i) being at least 16 years or older, (ii) self-reported injecting drug use within the past 12 months in the respective city, (iii) willing to take part in a questionnaire assisted-interview and to provide a capillary blood specimen for serological and molecular testing, (iv) giving informed consent, and (v) not having participated in the study previously. DBS samples from all 2,077 PWID enrolled in the study were investigated.

### Ethics statement

The Federal Commissioner for Data Protection and Freedom of Information approved the study protocol on 29/11/2012 (III-401/008#0035). Ethics approval was received on 19/11/2012 from the ethics committee at the medical university of Charité, Berlin (EA4/036/11) [39]. All adult subjects provided informed consent. No persons under the age of 18 were recruited and included in the study (although the minimum age for inclusion was 16 years). The median age of the participants within each of the eight cities varied from 29–41. Oral and written information on the study was provided to all potential participants. In most cases, participants signed thereafter a written consent form. In a limited number of cases, written consent was refused (for the reason of staying anonymous), but oral consent was obtained and given to the study site manager. In this case the study site manager signed the consent form in the presence of the participants as approved by the IRB.

## Results

### Validation of HTLV testing

**Fig 1:**
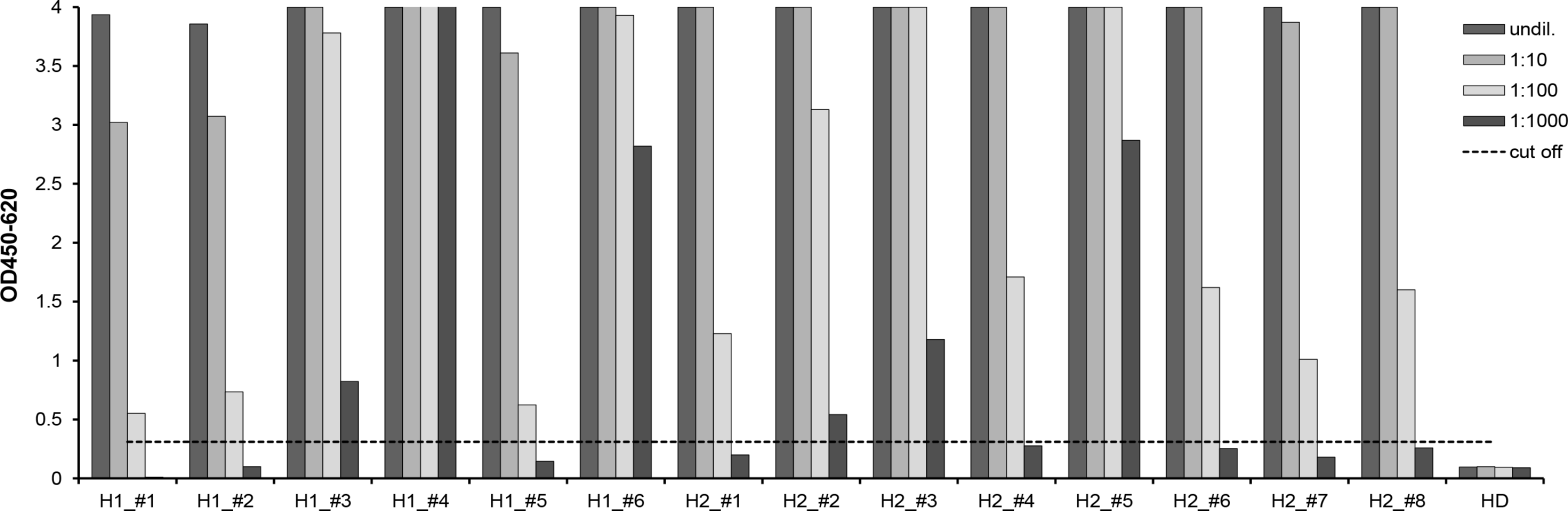
Sera tested in anti-HTLV-1/-2 ELISA. Serial dilutions of sera from six known HTLV-1 infected individuals (H1_#1 –H1_#6), eight HTLV-2 infected individuals (H2_#1 –H2_#8) and one healthy donor (HD) were tested with the commercial Murex HTLV-1/-2 ELISA. The cut-off (dashed line) was calculated as the mean OD of three replicates of the negative control normal human sera plus 0.2, according to the manufacturer's instructions.

To validate the use of DBS for HTLV screening, we tested sera from six known HTLV-1 infected individuals and eight HTLV-2 infected individuals with the diagnostic ELISA routinely used in our laboratory. In addition to the negative control sera provided with the assay, one serum from a healthy donor was included as control. To account for the probable different magnitudes of reactivity in individuals progressing to disease and asymptomatic carriers, serial dilutions up to 1:1000 were also tested. All HTLV-1 and HTLV-2 positive sera tested positive at the 1:100 dilution. Three HTLV-1 positive sera (H1_#3, H1_#4, H1_#6) and three HTLV-2 positive sera (H2_#2, H2_#3, H2_#5) were still positive at the 1:1000 dilution (Fig 1).

**Fig 2:**
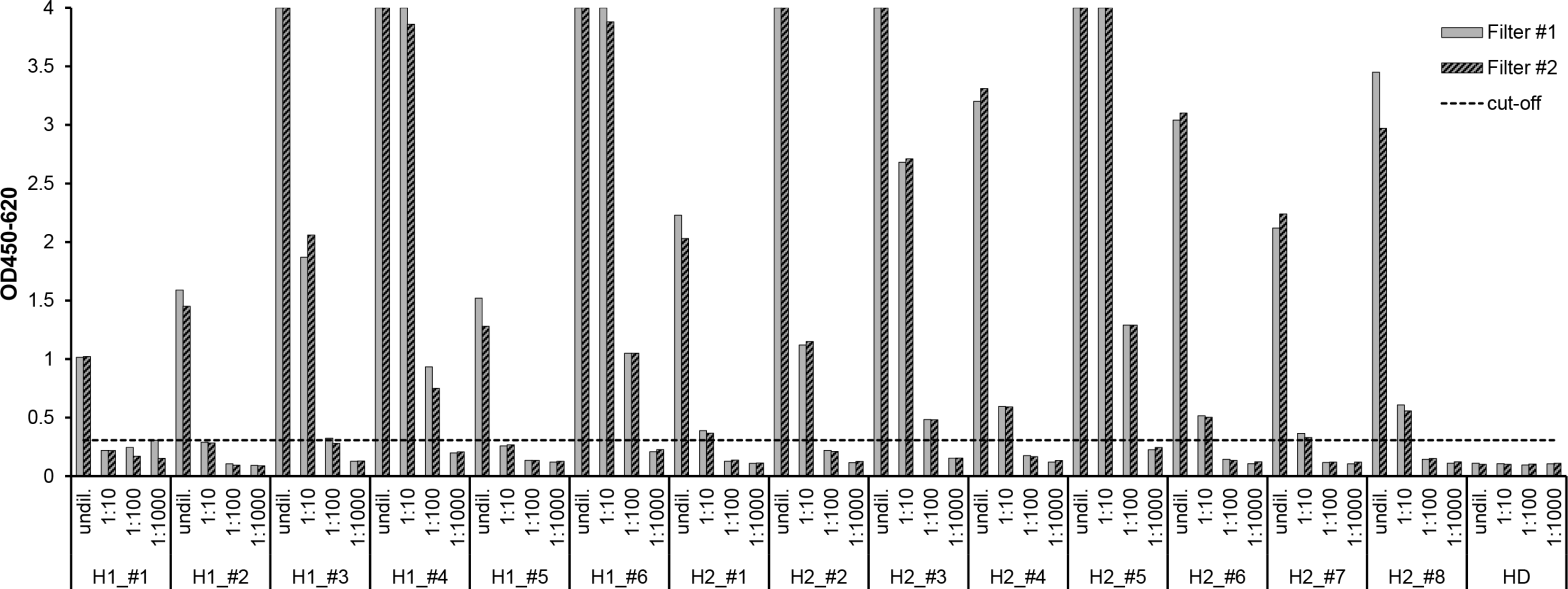
Elution of DSS tested in anti-HTLV-1/-2 ELISA. Serial dilutions of sera from six HTLV-1 infected individuals (H1_#1 –H1_#6), eight HTLV-2 infected individuals (H2_#1 –H2_#8) and one healthy donor (HD) were serially diluted and spotted on filter paper in duplicate (grey = eluate filter #1, hatched = eluate filter #2). Samples were eluted (resulting in an additional 1:15 dilution) and tested in the commercial Murex HTLV-1/-2 ELISA. The cut-off (dashed line) was calculated as the mean OD of three replicates of the negative control normal human sera plus 0.2, according to the manufacturer's instructions.

The same sera (and dilutions thereof) were then used to create DSS to simulate the conditions and processing used for the study samples. As expected, the inherent 1:15 dilution resulting from elution of the sera from the filters reduced detectability (Fig 2). All DSS made with undiluted sera from the 14 HTLV positive controls were reactive. Three samples (H1_#1, H1_#2, H1_#5) from the HTLV-1 panel (but none from the HTLV-2 panel) were already negative at the 1:10 pre-dilution. Two HTLV-1 positive samples (H1_#4, H1_#6) and two HTLV-2 positive samples (H2_#3, H2_#5) remained strongly reactive even at the 1:100 pre-dilution.

**Fig 3:**
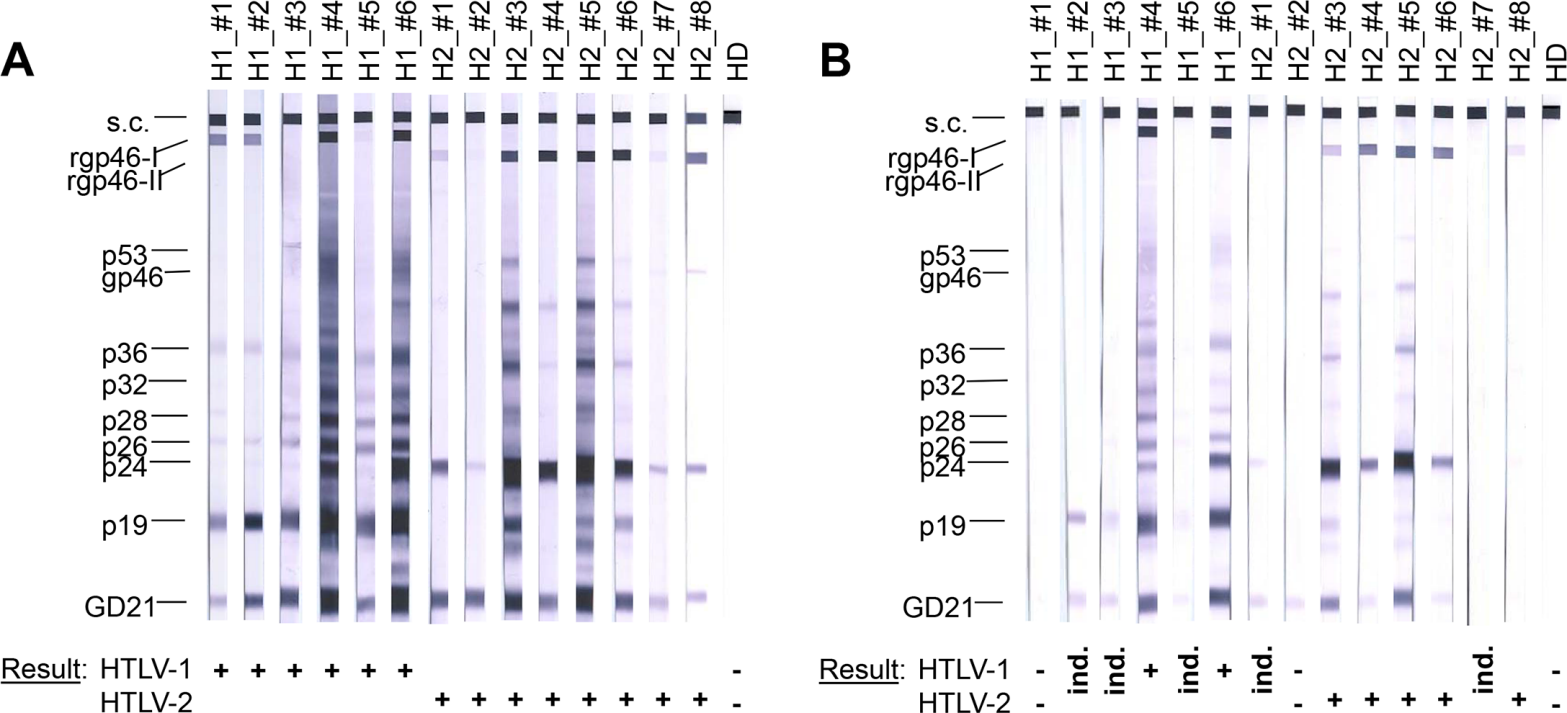
Use of Western blot for confirmation of HTLV infection in samples reactive in ELISA. (A) Sera from six HTLV-1 infected individuals (H1_#1 –H1_#6) and eight HTLV-2 infected individuals (H2_#1 –H2_#8) were tested in diagnostic Western blot and demonstrated varying reactivity to the envelope proteins (rgp46-I or rgp46-II, GD21) and the core proteins p19 and/or p24, plus sporadic reactivity to proteins such as p26, p28 and p36. A serum from a healthy donor (HD) was also tested as control. The top band (s.c., serum control) is an internal control to confirm addition of human sera to the nitrocellulose stripes. Typing (HTLV-1 or HTLV-2 infection) according to the manufacturer's guidelines is stated at the bottom. (B) Eluates from the dried serum spots for the corresponding sera were also tested by Western blot. Despite all samples being reactive by ELISA, not all eluates of the undiluted sera gave banding patterns in Western blot that confirm HTLV-1 or HTLV-2 infection. H1_#1 failed to react to any protein and H2_#2 reacted only to GD21 and both were therefore classified as negative. The eluates of H1_#2, H1_#3, H1_#5, H2_#1 and H2_#7 reacted to various proteins without fulfilling the criteria of confirmed HTLV infection, giving instead an indeterminate Western blot result. Sample H2_#7 was scored as indeterminate due to the faint bands for p24 and GD21. See Table 1 for detailed information about the banding patterns observed.

All sera were then subjected to diagnostic Western blot to test this assay's suitability for confirmation (Fig 3A). According to the manufacturer, a confirmed diagnosis requires reactivity to the two envelope proteins GD21 and rgp46-I or rgp46-II plus reactivity to one or both of the core antigens p19 and p24, or reactivity to both core antigens plus one of the envelope proteins. All but one sera fulfilled the criterion for HTLV-1 or HTLV-2 positivity (reactivity to GD21, rgp46-I or rgp46-II and p19 or p24), and other bands (e.g. p26, p28) were also sometimes visible at varying intensities. Only one serum (H1_#3) demonstrated reactivity to GD21, p19, p24 and other bands but not to rgp46-I or rgp46-II, although this could still be confirmed to be an HTLV-1 infection because the reactivity to p19 was stronger than that to p24.

After spotting on filter paper and eluting, only two HTLV-1 positive sera (H1_#4, H1_#6) remained unambiguously positive (Fig 3B, Table 1). Both remained positive even at a pre-dilution of 1:10, and one (H1_#6) was positive at 1:100 (Table 1). Five of the undiluted HTLV-2 positive sera (H2_#3 –H2_#6, H2_#8) were unambiguously positive after spotting and eluting, and three (H2_#3, H2_#5, H2_#6) remained positive at a pre-dilution of 1:10 (Table 1). Eluates of the undiluted HTLV-1 positive serum H1_#1 and HTLV-2 positive serum H2_#2, despite being positive in ELISA, tested negative by Western blot. The filter eluates of the three HTLV-1 positive sera H1_#2, H1_#3 and H1_#5 showed reactivity to a few core antigens including p19 and the GD21, which, according to the manufacturer, would be interpreted as an indeterminate HTLV-1/-2 Western blot result, but not as a confirmed HTLV infection. The filter eluates of two HTLV-2 positive sera (H2_#1, H2_#7) also showed reactivity to only the core antigen p24 and the GD21 antigen, resulting, as described above, in an indeterminate HTLV-1/-2 Western blot result. In contrast, for some of the sera, even the 1:10 pre-dilution could by typed by Western blot (H1_#4, H1_#6, H2_#3, H2_#5) and in one HTLV-1 positive serum H1_#6 the 1:100 pre-dilution could also be confirmed (Table 1).

**Table 1:** ELISA and confirmatory Western blot results of the validation.

ID	Sample	Dilution	ELISA reactive	Ratio^a^	Western blot HTLV type	Western blot bands
**H1_#1**						
	Serum	-	+	12.06	HTLV-1	rgp46-I, p36, p28, p26, p19, GD21
	Filter Eluate	-	+	3.1	-	-
	Filter Eluate	1:10	-		n.d.	
	Filter Eluate	1:100	-		n.d.	
	Filter Eluate	1:1000	-		n.d.	
**H1_#2**						
	Serum	-	+	11.8	HTLV-1	rgp46-I, p36, p26, p19, GD21
	Filter Eluate	-	+	4.7	indeterminate	p19, GD21
	Filter Eluate	1:10	-		n.d.	
	Filter Eluate	1:100	-		n.d.	
	Filter Eluate	1:1000	-		n.d.	
**H1_#3**						
	Serum	-	+	>12.5^b^	HTLV-1	p36, p28, p26, p24, p19, GD21
	Filter Eluate	-	+	>12.5^b^	indeterminate	p26, p19, GD21
	Filter Eluate	1:10	+	6.9	-	-
	Filter Eluate	1:100	-		n.d.	
	Filter Eluate	1:1000	-		n.d.	
**H1_#4**						
	Serum	-	+	>12.5^b^	HTLV-1	rgp46-I, p53, gp46, p36, p32, p28, p26, p24, p19, GD21
	Filter Eluate	-	+	>12.5^b^	HTLV-1	rgp46-I, p53, gp46, p36, p32, p28, p26, p24, p19, GD21
	Filter Eluate	1:10	+	>12.5^b^	HTLV-1	rgp46-I, p36, p32, p28, p26, p24, p19, GD21
	Filter Eluate	1:100	+	2.9	-	rgp46-I
	Filter Eluate	1:1000	-		n.d.	
**H1_#5**						
	Serum	-	+	>12.5^b^	HTLV-1	rgp46-I, p36, p32, p28, p26, p24, p19, GD21
	Filter Eluate	-	+	4.0	indeterminate	p28, p26, p19, GD21
	Filter Eluate	1:10	-		n.d.	
	Filter Eluate	1:100	-		n.d.	
	Filter Eluate	1:1000	-		n.d.	
**H1_#6**						
	Serum	-	+	>12.5^b^	HTLV-1	rgp46-I, p53, gp46, p36, p32, p28, p26, p24, p19, GD21
	Filter Eluate	-	+	>12.5^b^	HTLV-1	rgp46-I, p53, gp46, p36, p32, p28, p26, p24, p19, GD21
	Filter Eluate	1:10	+	12.4	HTLV-1	rgp46-I, p36, p24, p19, GD21
	Filter Eluate	1:100	+	3.3	HTLV-1	rgp46-I, p24, p19, GD21
	Filter Eluate	1:1000	-		n.d.	
**H2_#1**						
	Serum	-	+	>12.5^b^	HTLV-2	rgp46-II, p24, GD21
	Filter Eluate	-	+	7.8	indeterminate	p24, GD21
	Filter Eluate	1:10	+	1.4	-	p24
	Filter Eluate	1:100	-		n.d.	
	Filter Eluate	1:1000	-		n.d.	
**H2_#2**						
	Serum	-	+	>12.5^b^	HTLV-2	rgp46-II, p24, GD21
	Filter Eluate	-	+	>12.5^b^	-	GD21
	Filter Eluate	1:10	+	3.9	-	-
	Filter Eluate	1:100	-		n.d.	
	Filter Eluate	1:1000	-		n.d.	
**H2_#3**						
	Serum	-	+	>12.5^b^	HTLV-2	rgp46-II, gp46, p36, p28, p26, p24, p19, GD21
	Filter Eluate	-	+	>12.5^b^	HTLV-2	rgp46-II, p53, p36, p24, p19, GD21
	Filter Eluate	1:10	+	8.5	HTLV-2	rgp46-II, p24, p19, GD21
	Filter Eluate	1:100	+	1.5	-	p24
	Filter Eluate	1:1000	-		n.d.	
**H2_#4**						
	Serum	-	+	>12.5^b^	HTLV-2	rgp46-II, p36, p24, GD21
	Filter Eluate	-	+	10.2	HTLV-2	rgp46-II, p24, GD21
	Filter Eluate	1:10	+	1.9	indeterminate	rgp46-II, p24
	Filter Eluate	1:100	-		n.d.	
	Filter Eluate	1:1000	-		n.d.	
**H2_#5**						
	Serum	-	+	>12.5^b^	HTLV-2	rgp46-II, p53, gp46, p36, p28, p24, p19, GD21
	Filter Eluate	-	+	>12.5^b^	HTLV-2	rgp46-II, p53, p36, p28, p24, p19, GD21
	Filter Eluate	1:10	+	>12.5^b^	HTLV-2	rgp46-II, p36, p24, GD21
	Filter Eluate	1:100	+	4.1	indeterminate	rgp46-II, p24
	Filter Eluate	1:1000	-		n.d.	
**H2_#6**						
	Serum	-	+	>12.5^b^	HTLV-2	rgp46-II, p36, p28, p24, p19, GD21
	Filter Eluate	-	+	9.7	HTLV-2	rgp46-II, p24, p19, GD21
	Filter Eluate	1:10	+	1.6	indeterminate	rgp46-II, p24
	Filter Eluate	1:100	-		n.d.	
	Filter Eluate	1:1000	-		n.d.	
**H2_#7**						
	Serum	-	+	>12.5^b^	HTLV-2	rgp46-II, p24, GD21
	Filter Eluate	-	+	6.9	indeterminate	p24, GD21
	Filter Eluate	1:10	+	1.1	-	-
	Filter Eluate	1:100	-		n.d.	
	Filter Eluate	1:1000	-		n.d.	
**H2_#8**						
	Serum	-	+	>12.5^b^	HTLV-2	rgp46-II, p24, p19, GD21
	Filter Eluate	-	+	10.2	HTLV-2	rgp46-II, p24, GD21
	Filter Eluate	1:10	+	1.9	-	-
	Filter Eluate	1:100	-		n.d.	
	Filter Eluate	1:1000	-		n.d.	

n.d., not done.

^a^ Ratio (sample OD/cut-off OD), mean of two processed filter.

^b^ above detection limit.

Taken together, all DSS eluates of the validation panel were found to be positive in ELISA, but testing eluates of six DSS from HTLV-1 infected asymptomatic carriers resulted in only two cases that could be confirmed by Western blot. In contrast, testing eight DSS from HTLV-2 infected asymptomatic carriers resulted in five confirmed HTLV-2 infections. A summary of the validation, with details of the ELISA and Western blot results, is given in Table 1. In conclusion, these data indicate that even for HTLV-infected individuals with low antibody titers, eluates from DBS can still test positive by ELISA, although a venous blood sample appears in a few cases to be necessary for confirmation.

### Determination of HTLV prevalence among PWID in Germany

Initial screening of the DBS eluates was performed by ELISA as described above. Of the 2,077 eluates from DBS tested, six were initially found to be reactive (Table 2). Three were only weakly reactive with a sample/cut-off ratio of <2, and three were stronger with ratios of 2.7, 5.0 and 5.8. The sample/cut-off ratios of one strong positive HTLV-1 serum and the internal positive control which was included on every assay plate were both >12.5. The six samples were tested again in duplicate in a second ELISA assay and found to be negative. Thus, no HTLV infections were confirmed in our large study population of German PWID.

**Table 2:** Determination of HTLV prevalence among PWID in Germany.

Samples	1^st^ screening ELISA			2^nd^ ELISA test	WB	
	Non-reactive	Weak reactive, ratio^a^ 1–1.9	Strong reactive, ratio^a^ >2	Non- reactive		
2077	2071	3	3	6	n.d.	

^a^ratio, (sample OD/cut-off OD)

n.d., not done.

## Discussion

HIV and HTLV are the only known exogenous human retroviruses, and of the four types of HTLV, only HTLV-1 and HTLV-2 are of epidemiological relevance, both being distributed worldwide, albeit usually in different population groups. Both viruses can spread easily within people who inject drugs (PWID), with especially HTLV-2 being found at high rates in PWID within the United States and Europe [12].

Here we report the results of screening for anti-HTLV-1/-2 antibodies in 2,077 samples from German PWID collected during 2011–2014 as part of a multicenter sero-behavioural survey using respondent-driven sampling [39]. The frequent sharing of needles and equipment by PWID place them at particularly high-risk for blood-borne diseases such as HIV, hepatitis and also HTLV. Studies in other European countries found varying seroprevalences of HTLV-1 and -2 among PWID [40, 42–48]. Although the prevalences for hepatitis B and C and HIV in German PWID are comparable to those in other European countries and are much higher than in the general population [41], we found no case of HTLV-1 or -2 infection in our study population. This finding is in line with an older study [38] carried out in a small sample of 63 German PWID that also failed to find a single case of HTLV infection and, in addition, reflects the absence or very low prevalence of HTLV infection in the general population. In German blood donors and pregnant women, the proportion of HTLV infection was found to be 0 and 0.7 per 10,000, respectively [35, 38].

Our study was performed with DBS material prepared from capillary blood. The use of DBS samples for HTLV seroprevalence studies is not without precedence. In one Spanish study, DBS from 484 PWID were analyzed and 27 cases of HTLV-2 infection were detected [40], using both Western blot and PCR for confirmation. It is not clear from the paper how many samples needed to be confirmed using PCR due to having titers too low for Western blot confirmation. There are also older data available addressing the use of DBS for the detection of anti-HTLV antibodies, one in which simulated HTLV-1 positive DBS prepared using sera from HAM/TSP or ATL patients were evaluated [31] and another carried out in the Gauteng region of South Africa in which 2,582 DBS from neonates were screened and 10 cases of HTLV infection were found [36]. Both studies used the same procedure for testing the filter eluates, i.e. HTLV-I gelatin particle agglutination for screening followed by confirmation by Western blot. However, in these studies antibodies were extracted into a smaller volume from only a portion of the DBS, and the wide range of antibody titers in HTLV-infected individuals makes it difficult to evaluate the impact of such variations in extraction methods on the subsequent Western blot result. A large prospective study of pregnant women in Europe, also used DBS samples from France, England and Germany [35], and it is worthwhile to note that of the 104 samples repeatedly reactive by particle agglutination test or ELISA, only 83 could be confirmed by Western blot or Line Immuno-Assay. Furthermore, a recent study comparing 692 DBS and their corresponding venous blood samples for the serological detection of several infections, found that both methods successfully detected several cases, including one HTLV infection [29], although no confirmation assay was used. Finally, one large study analyzed 55,293 DBS for HTLV seroprevalence, and although Western blot was not used for confirmation, it was possible to investigate the corresponding blood samples for confirmation [34]. Despite the frequent use of DBS in earlier HTLV seroprevalence studies mentioned above, our validation results using serial dilutions of 14 positive sera indicate an increased risk of missing positive samples that have exceptionally low antibody levels if DBS are used in place of regular serum or plasma for ELISA screening. One study using simulated dried blood spots with sera or plasma from HAM/TSP or ATL patients found only two filter eluates samples giving indeterminate results in Western blot confirmatory testing, whereas 24 samples could be positively confirmed [31]. However, here the authors also found the antibody titers in filter eluates measured with the HTLV-1 gelatin particle agglutination test to be drastically reduced compared to the titers of the original sera or plasma samples. The frequency of HTLV infected persons with such critical antibody levels is presumably low but needs to be determined far more precisely in order to reliably estimate the limitations imposed upon DBS usage for HTLV surveillance studies in the future. Furthermore, the results obtained show that a direct application of the DBS material obtained using a procedure such as ours or similar for Western blot confirmation of an HTLV infection is not reliable and should rather be performed with regular serum or plasma or by PCR.

The prevalence of HTLV in PWID is quite different across European countries. In the Irish city of Dublin, a prevalence of 14.6% HTLV-2 seropositivity in a sample of 103 PWID infected with HIV-1 was reported, but no HTLV-1. In this study, sera were tested by ELISA and infections were confirmed by Western blot and/or PCR [43] using the same commercial assays used in our study (Murex HTLV I+II ELISA; MP Diagnostics HTLV Blot 2.4). In contrast, in a sample of 583 Portuguese PWID infected with HIV-1 a low seroprevalence of 0.51% was observed, and again only HTLV-2. The sera were tested by ELISA and Western blot using the same assays as in our study, and one of the three ELISA positive samples remained indeterminate in Western blot [46]. It is worthwhile to note that each of the infected individuals reported to have lived in Spain for long periods of time and that the phylogenetic analysis of the LTR sequences from their viruses suggested a Spanish origin. In Madrid and Barcelona, relatively high prevalences of HTLV-2, 17/256 (6.6%) and 10/228 (4.4%) respectively, have been reported in PWID recruited via respondent-driven sampling [40]. It is notable that in this study no HTLV infections were detected in PWID recruited in a third Spanish city, Seville. This study also used DBS collected over a period of nearly three years and all cases were identified by ELISA and confirmed by Western blot (and/or PCR) using the same kits used in our study. Furthermore, according to one brief report [48], unpublished data suggest an even higher prevalence in Spanish PWID coinfected with HIV-1, with 67/695 (9.65%) being infected, although none of the 432 HIV-1 negative, HCV positive members of the study participants in the same hospital tested positive for HTLV. A large study conducted in 3,574 PWID in Italy from 1986 to 2005 revealed different prevalences of HTLV infection in HIV-1 coinfected (6.7%) and HIV-1 negative individuals (1.1%) [47]. Serum or plasma samples in this study were first screened by passive particle agglutination (Fujirebio, Tokyo, Japan), and confirmation (as well as HTLV-1/HTLV-2 classification) was performed using the same ELISA and Western blot kits used in our study. Interestingly, despite the PWID study participants being recruited from locations throughout Italy, all infections were classified as HTLV-2. In a more recent report using samples from group of Swedish PWIDs, the ARCHITECT ® rHTLV-I/II system was used for screening and the INNOLIA™ HTLVI/II score assay for confirmation [45], and an HTLV seroprevalence of 35/1079 (3.2%) was found. Again, the majority of HTLV infections were attributed to HTLV-2, with only two scoring positive for HTLV-1 and another five as indeterminate. Finally, a very recent study carried out in Tallinn, Estonia, with 345 PWID recruited by respondent-driven sampling [44] found only one case of HTLV-2 infection. In this case PBMC DNA was directly analyzed by nested PCR and no serological assays were used.

It is currently assumed that at least two different introductions of HTLV-2 from the US into PWID populations in Europe took place, based on the observation that the HTLV-2a subtype is found predominantly in Northern and Eastern Europe whereas the HTLV-2b subtype is mainly found in Western and Southern Europe [7, 42]. The highly variable HTLV prevalences in PWID from different European countries or even within different cities in one country, including the zero prevalence among German PWID reported here, is quite puzzling. Furthermore the prevalences of HTLV in PWID seem to be very stable: for example in Spanish PWID, HTLV-2 but no HTLV-1 was found despite the increased proportions of migrants from regions in which HTLV-1 is more prevalent [40]. Furthermore, the most recent study of HTLV seroprevalence in Stockholm found it to be comparable to previous studies from the early 1990s [45]. Moreover, the fact that HTLV-2 is predominately found in PWID [40, 42–48], even in countries where HTLV-1 is found in the general population [35], suggests a distinct founder events for HTLV infection in the various PWID groups studied so far.

In conclusion, this analysis of 2,077 DBS samples from German PWID recruited by respondent-driven sampling in eight German cities between 2011 and 2014 found no evidence for cases of HTLV infection. These results are in line with previous serological surveys carried out in Germany but are somewhat different from the situation in some other European countries where low HTLV seroprevalences have been reported, particularly in PWIDs.
